# Supplementary material for: The interactome of intact mitochondria by cross-linking mass spectrometry provides evidence for coexisting respiratory supercomplexes
Source: Mol Cell Proteomics. 2017 Dec 8;17(2):216–32. doi: 10.1074/mcp.RA117.000470 (PMC5795388; doi:10.1074/mcp.RA117.000470)
Supplement: Supplemental Data [file supp_RA117.000470_133922_0_supp_23978_fzffwf.zip › spectra_annotation/mito_DR_spectra_annotation/194-1-5-1-3-1.pdf]

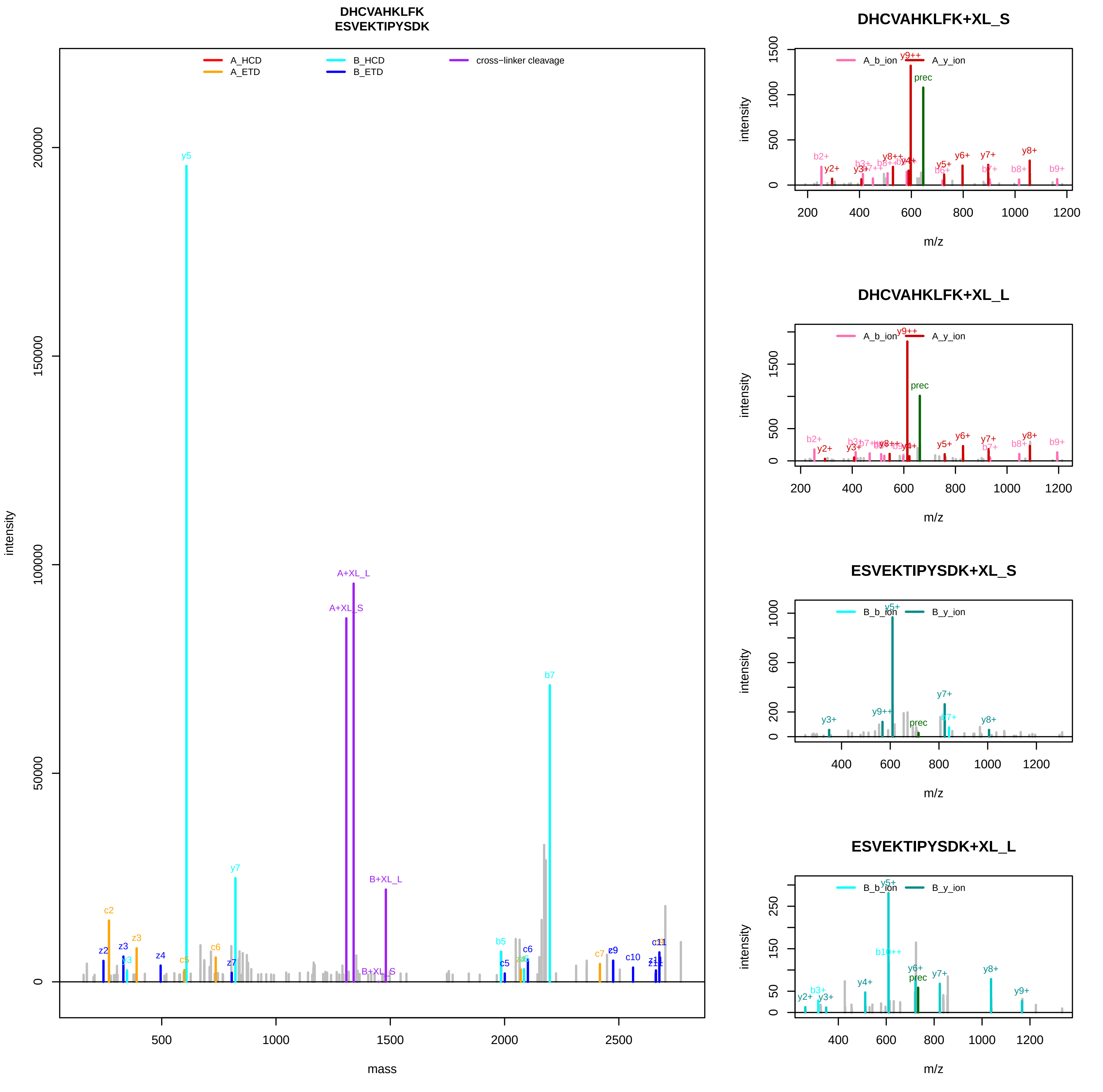

DHCVAHKLFK
ESVEKTIPYSDK
A_HCD
A_ETD
B_HCD
B_ETD
cross−linker cleavage
intensity
mass
y5
y7
b5
b7
A+XL_L
A+XL_S
B+XL_L
B+XL_S
c2
c5
c6
c7
c10
c11
z2
z3
z4
z7
z9
c9
DHCVAHKLFK+XL_S
DHCVAHKLFK+XL_L
ESVEKTIPYSDK+XL_S
ESVEKTIPYSDK+XL_L
A_b_ion
A_y_ion
B_b_ion
B_y_ion
prec
m/z
y9++
y8+
y7+
y6+
y5+
y4+
y3+
y2+
b2+
b3+
b7+
b8+
b9+
y9+
y8++
y9++
b10++
